# Creation and resistance evaluation of a new soybean germplasm rich in betalain

**DOI:** 10.3389/fpls.2025.1743684

**Published:** 2026-01-19

**Authors:** Yuwei Bi, Qi Zhang, Yun He, Yi Ding, Haoran Tian, Fan Zeng, Kaidi Lyu, Haiyun Li, Shanhua Lyu, Yinglun Fan

**Affiliations:** College of Agriculture and Life Science, Liaocheng University, Liaocheng, China

**Keywords:** betalain, *Bradyrhizobium japonicum*, brown planthopper, plant-microbe interaction, *RUBY* reporter system, soybean (glycine max)

## Abstract

The betalain biosynthesis system (*RUBY*) exhibits a stable cross-species coloration advantage in plant genetic transformation. As a visually detectable genetic marker (visible to the naked eye), the color marker holds enormous application potential in positive selection of transgenic plants, identification of hybrids between different plant varieties, haploid selection, and other research. However, when applying the *RUBY* to plant-microbe interaction research, it is necessary to clarify whether the biosynthesis of betalain and its accumulation in plant tissues and organs alter the plant-microbe interaction processes, including symbiotic or antagonistic relationships. In this study, *RUBY* transgenic soybean was created. There were no significant differences in nodule number, fresh weight, and dry weight of nodule between the *RUBY* transgenic line and wildtype soybean after inoculation with *Bradyrhizobium japonicum*. The biosynthesis and accumulation of betalain did not affect the infection and colonization of rhizobia. The *RUBY* transgenic line and wildtype soybean were inoculated with *Phomopsis longicolla*. The results showed that the biosynthesis and accumulation of betalain did not alter the infection and spread of *P. longicolla*. In field experiments, investigations found that the number of adult brown planthoppers and their eggs attached to the leaves of the *RUBY* transgenic line was extremely significantly lower than that of the wildtype soybean. This indicates that betalain accumulation may endow soybean with a repellent effect against herbivorous insects. This work revealed that the heterologous biosynthesis and accumulation of betalain in soybean neither affect the nodulation ability of soybean with rhizobia, nor interfere with the infection of soybean by pathogenic bacteria, but also reduce the damage caused by brown planthoppers to soybean. Analysis of the field investigation data on agronomic traits indicated that transgenic soybeans with low betalain content, exerted no adverse effects. In contrast, the transgenic soybean with high betalain content, exhibited negative impacts on node number on main stem, plant height, and yield.

## Introduction

In the process of plant genetic transformation, selectable or reporter genes are essential for distinguishing transgenic events from non-transgenic events (Nishizawa et al., 2006; Koo et al., 2007; He et al., 2020). During stable genetic transformation, antibiotic or herbicide resistance genes need to be added for the screening of positive calli. For instance, in the genetic transformation of soybean, the bialaphos resistance (*Bar*) gene is commonly used to screen positive calli. However, after the obtained positive plants undergo self-breeding in the next generation, PCR or Bar strip test detection is usually necessary to distinguish transgenic plants from non-transgenic plants. In recent years, the developed visual reporter genes have provided a highly convenient selection method for the screening of transgenic plants in the current generation or their progeny (Chiu et al., 1996; Nishizawa et al., 2006; Lin et al., 2011; Fan et al., 2020; He et al., 2020). Betalain is a natural pigment with a nitrogen-containing heterocyclic structure. It accumulates in plants such as Beetroot (*Beta vulgaris* subsp. vulgaris) and Red Dragon Fruit (*Hylocereus polyrhizus*), giving these plants a vibrant color (Polturak and Aharoni, 2019). The biosynthesis pathway of betalain is regulated by three key enzymes, namely cytochrome P450 enzyme, L-DOPA 4,5-dioxygenase, and glucosyltransferase. When the three genes were co-expressed under the same open reading frame (named *RUBY*), the synthesis and accumulation of betalain can be achieved in *RUBY* transgenic positive plants, endowing the organs of the transgenic plants with a vivid red color (He et al., 2020; Polturak and Aharoni, 2019). When the *RUBY* reporter system was applied to the genetic transformation of plants such as soybean, tomato, rice, *Arabidopsis thaliana*, carrot, and pepper, the accumulation of betalain in different tissues and organs of these plants became visible to the naked eye (He et al., 2020; Grützner et al., 2021; Wang et al., 2023; Chen et al., 2024; Tan et al., 2025; Tang et al., 2025). When the *RUBY* was used as a reporter system, the synthesis and accumulation of betalain in the plants did not affect callus induction, plant regeneration, development, or fertility (Yu et al., 2023). Naked-eye visual genetic markers have undeniable advantages in plant genetic transformation, as they can easily identify transgenic events from non-transgenic events. In particular, naked-eye visual color markers have great application potential in the identification of hybrids between plant varieties and the screening of haploids (Wang et al., 2023). The visualization of betalain can also realize real-time monitoring of gene expression. For example, in the study of tracking the colonization of arbuscular mycorrhizal (AM) fungi in plant roots, an inducible promoter was used to drive the expression of *RUBY*, so that the color of betalain is only induced in root tissues and cells colonized by fungi, realizing the accurate marking of colonized areas (Timoneda et al., 2021). The application of the *RUBY* reporter system in the CRISPR editing system enables the rapid screening of mutants without exogenous genes through cotyledon color in the early seed germination stage (Chen et al., 2024). By combining *RUBY* with CRISPR editing technology, all gene editing plants showed red color in T0 generation plants and 100% of the progeny plants did not carry any transgenic components in the T1 generation (Zhu et al., 2025a).

Soybean (*Glycine max* (L.) Merrill) is one of the globally essential oil crops. Its economic value is mainly reflected in providing dietary protein and vegetable oils for humans, and it can also be used as animal feed, biomaterials, processing raw materials, etc (Hartman et al., 2016). The interaction between soybean and environmental microorganisms is dualistic: the symbiotic mutualism effect can directly promote their own growth, while the pathogenic antagonism effect can indirectly ensure growth safety (Pineda et al., 2013). On the one hand, rhizobia provide 50%-97% of the nitrogen source for soybean through symbiotic nitrogen fixation (Mohammed and Dakora, 2024), and their nodulation efficiency directly affects crop yield and nitrogen fertilizer demand (Pandey et al., 2022). For instance, field experiments conducted in nutrient-deficient soils, such as those in Ethiopia and southwestern Iran, have confirmed that rhizobial inoculation can significantly enhance the yield of common beans (Samago et al., 2018). On the other hand, many pathogenic bacteria can cause soybean diseases. For instance, *Phomopsis longicolla* is a key seed-borne fungal pathogen and the primary causal agent of Phomopsis seed decay (PSD) in soybean. As one of the most devastating seed diseases worldwide, PSD severely impairs soybean seed quality and causes substantial yield losses, resulting in severe economic losses (Jing et al., 2018; Olszak-Przybyś and Korbecka-Glinka, 2024). During the growth and development process, to cope with these threats, soybean has evolved various defense mechanisms, and the synthesis of secondary metabolites is one of the measures. Plant secondary metabolites can enhance the stress resistance of plants and prevent the infection and spread of pathogenic bacteria [24]. Melatonin could enhance the resistance to stripe rust in wheat (Jiang et al., 2025). However, as a secondary metabolite, it remains unclear whether the accumulation of betalain in plant organs affects the interaction between plants and microorganisms. Plant secondary metabolites also play an essential role in defending against herbivorous insects. Grain amaranth (*Amaranthus hypochondriacus*) exhibits enhanced resistance to insect herbivory, which correlates with higher betalain pigmentation (Portillo-Nava et al., 2021). When the larvae of tobacco hornworm (*Manduca sexta*) feed on red-leaf amaranth (*Amaranthus cruentus*) or consume artificial diets supplemented with red-leaf pigment extracts (betalain pigmentation), their growth, development, and vitality are all affected more severely. The underlying mechanism for this phenomenon is maybe as follows: the enhancement of plant resistance to insect herbivory mediated by betalain accumulation may be attributed to the reduced digestibility of plant nutrients, particularly proteins, caused by their covalent interaction with oxidized betalain derivatives. These derivatives are synchronously generated during the process in which chewing insect infestation induces the upregulation of polyphenol oxidase activity; another contributing mechanism is that the vibrant pigmentation inherent to betalains can act as an aposematic signal, thereby conferring protection on plants against attacks by herbivorous organisms (Portillo-Nava et al., 2021). These reports suggest that betalain may act as a broad-spectrum insect-resistant signal or repellent substance. Betalains exhibit significant protective functions, specifically defending plants against abiotic stresses as well as various pathogens and viruses (Jin et al., 2025).

When the *RUBY* system was used as a reporter to investigate the interaction between soybean and microorganisms, we could not yet determine whether the accumulation of betalain would affect the interaction process between soybean and microorganisms. It is well known that symbiotic nitrogen fixation between soybean and rhizobia constitutes a crucial pathway for soybean to acquire nitrogen. In contrast, *Phomopsis longicolla* (the causal agent of Phomopsis seed decay) severely impairs soybean yield. In this study, we selected these two microorganisms (one beneficial and one pathogenic) as model systems to explore whether betalain accumulation would interfere with the soybean-microbe interactions. Additionally, it intended to investigate the effects of betalain accumulation in soybean resistance to herbivorous insects and the agronomic traits of transgenic soybean.

## Results

### Construction of *RUBY* expression vector and acquisition of transgenic lines

The *RUBY* expression cassette was cloned between *Nco*I and *Pml*I of the pCAMBIA3301 vector and driven by CaMV 35S promoter. The successfully constructed vector was named pB35RUBY (**Supplementary Figure S1**). After transforming the soybean cultivar Williams82 with *A. tumefaciens* strain EHA105 harboring the plasmid pB35RUBY, a total of 11 transgenic plants resistant to the glufosinate herbicide were obtained, among which the leaves of nine T0 transgenic lines exhibited a red phenotype (**Supplementary Figure S2**).

**Figure 1:**
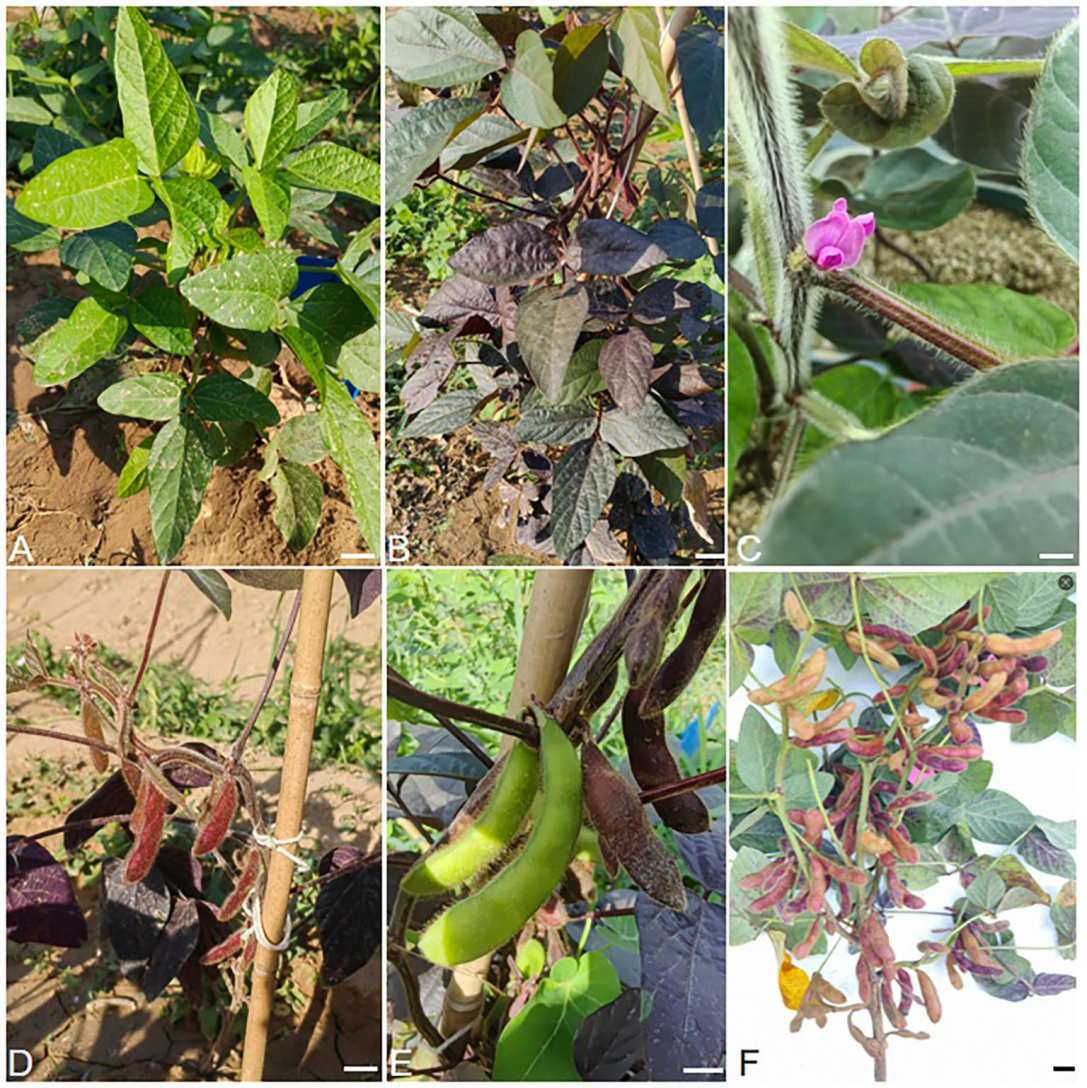
Field performance of *RUBY* genetically modified soybean. **(A)** wildtype Williams82. **(B)** R1 (Soybean developmental stage of beginning bloom) stage of Rline2. **(C)** the flower of Rline2. **(D)** R4 (Soybean developmental stage of early pod fill) stage of Rline1. **(E)** R5 (Soybean developmental stage of beginning seed) stage of Rline5. **(F)** R7 (Soybean developmental stage of initial ripening stage) of Rline7. Bars=1 cm.

The nine red colored T0 transgenic lines were planted in the field (**Figures 1A–F**). During the flowering stage, all these transgenic plants exhibited red-colored flowers (**Figure 1C**), and red pods (**Figure 1D**). Among them, one line, RLine5, showed a chimeric phenotype, with its pods being both red and green (**Figure 1E**). Another line, RLine7, had pods with significant variations in color intensity (**Figure 1F**).

**Figure 2:**
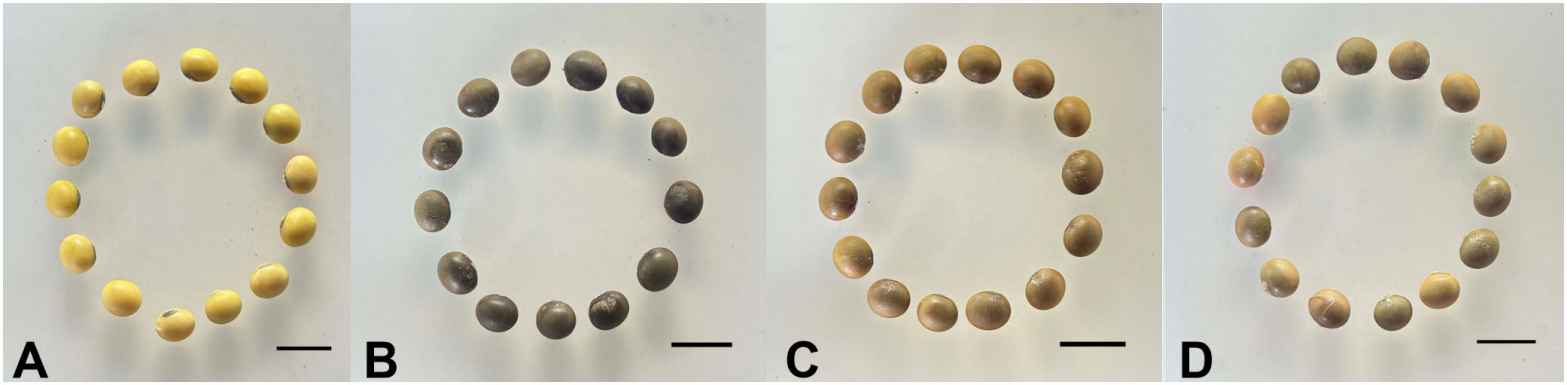
The seeds of soybean. **(A)** wildtype Williams82. **(B–D)**, RLine1, RLine2, and RLine6, respectively. Bars=1 cm.

Self-pollination of the nine T0 transgenic lines yielded T1 generation transgenic soybean seeds. Subsequent field planting of the T1 seeds revealed that three of the transgenic lines (RLine4, 5, and 7) no longer showed red coloration in their leaves, stems, or the entire plant, indicating the absence of betalain accumulation. We inferred that these three lines were either chimeras or that transgene silencing had occurred, resulting in the failing of *RUBY* expression. The remaining six T1 lines were self-pollinated, and T2 generation soybean seeds were harvested. Through the whole plant color screening, three transgenic lines (RLine1, RLine2, and RLine6) were identified, which had a homozygous *RUBY* genotype, genetic stability, and stable expression of the *RUBY*. These three lines were used in the experiments of this study. Among these lines, RLine1 exhibited a deep red coloration across the whole plant, with its roots also showing a bright red hue. The color of the other two lines, RLine2 and RLine6, was lighter than that of RLine1, and the T2 generation seeds of these three lines exhibited brown coloration at varying intensities (**Figure 2**).

### Effect of synthesis and accumulation of betalain on the colonization of *Rhizobia*

**Figure 3:**
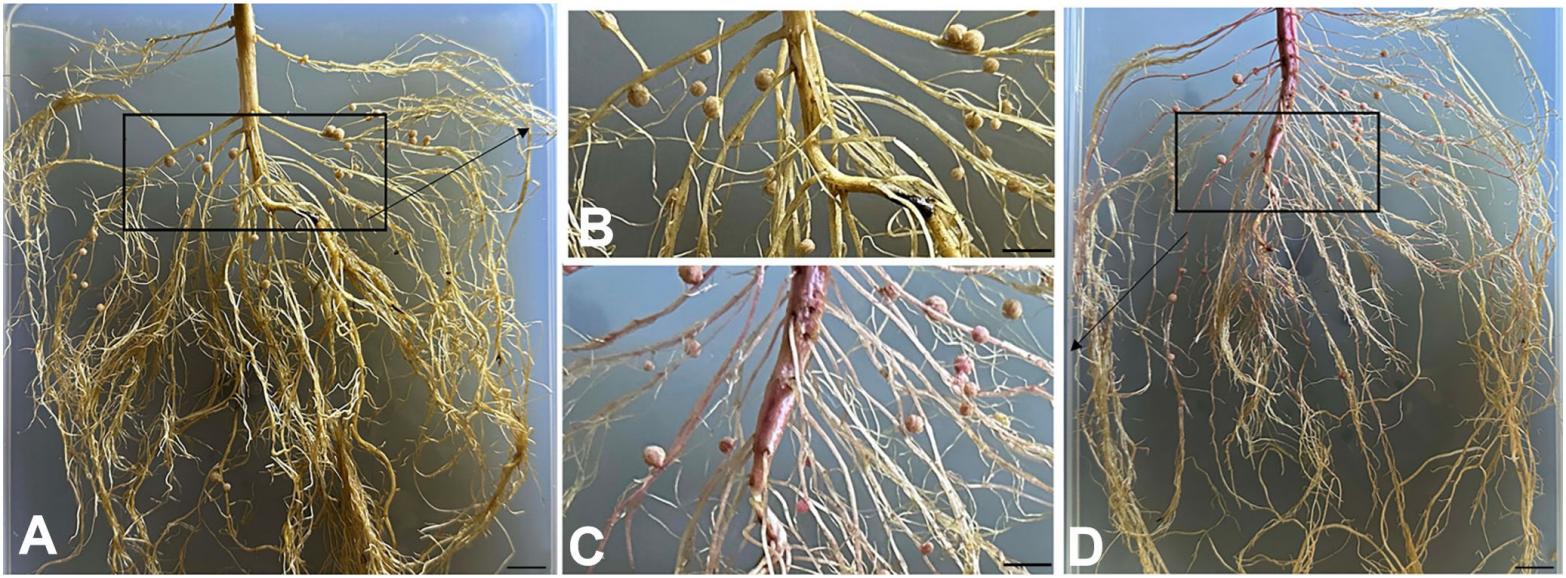
Evaluate the nodation ability of soybean and rhizoma. **(A, B)** wildtype Williams82. **(C, D)** RLine1 Bars=1 cm.

**Figure 4:**
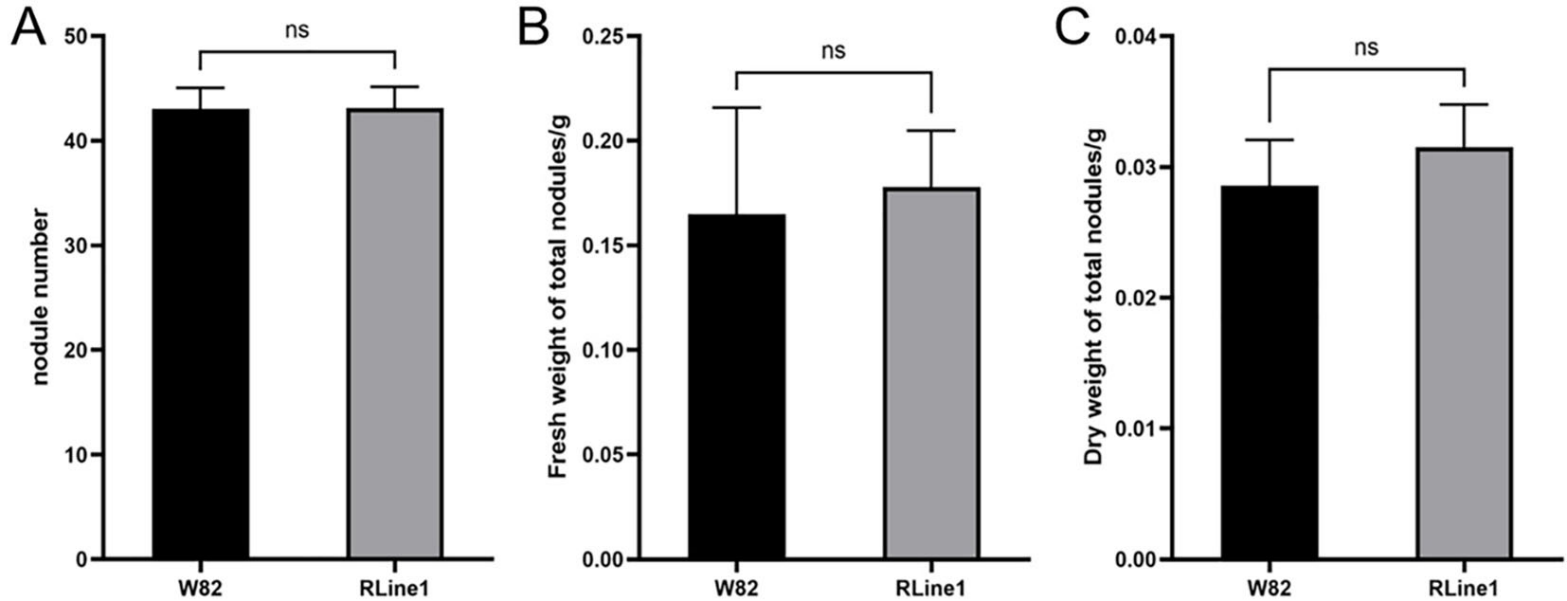
The differences in nodule number **(A)**, fresh weight **(B)**, and dry weight **(C)** between the betalain-enriched RLine1 line and the wildtype Williams82. ‘ns’ indicates that there is no statistically significant difference between the compared data. Data are the mean ± SD with ten biological replicates. p>0.05, (*t*-test).

Plants of the RLine1 line exhibited an intense deep-red phenotype throughout the whole plant body, with high betalain accumulation also detected in the roots. In the rhizobium inoculation assay, the RLine1 line and the wildtype line Willams82 we employed. After 28 d of inoculating the transgenic soybean line RLine1 and the wildtype soybean Williams82 with *B. japonicum* USDA110, the growth of root nodules was investigated (**Figure 3**). In terms of the number of nodules formed in soybean roots, the average nodule counts of the transgenic soybean line RLine1 and the wildtype soybean Williams82 were 43.0 and 43.1, respectively, with no significant difference. This result suggests that the accumulation of betalain in roots did not affect the infection and colonization of rhizobia. The corresponding fresh weights of root nodules on the two soybean lines, wildtype Williams82 and transgenic line RLine1, were 0.1649 g and 0.1779 g, respectively, while their dry weights were 0.0286 g and 0.0315 g, respectively. Statistically, no significant differences were detected in either the fresh or dry weights of root nodules between the two lines; however, the average values of the transgenic line RLine1 were slightly higher (**Figure 4**). The results indicated that the accumulation of betacyanin in soybean roots did not affect the infection and colonization of rhizobia.

### Effect of betalain accumulation on the infection of soybean by *P. longicolla*

**Figure 5:**
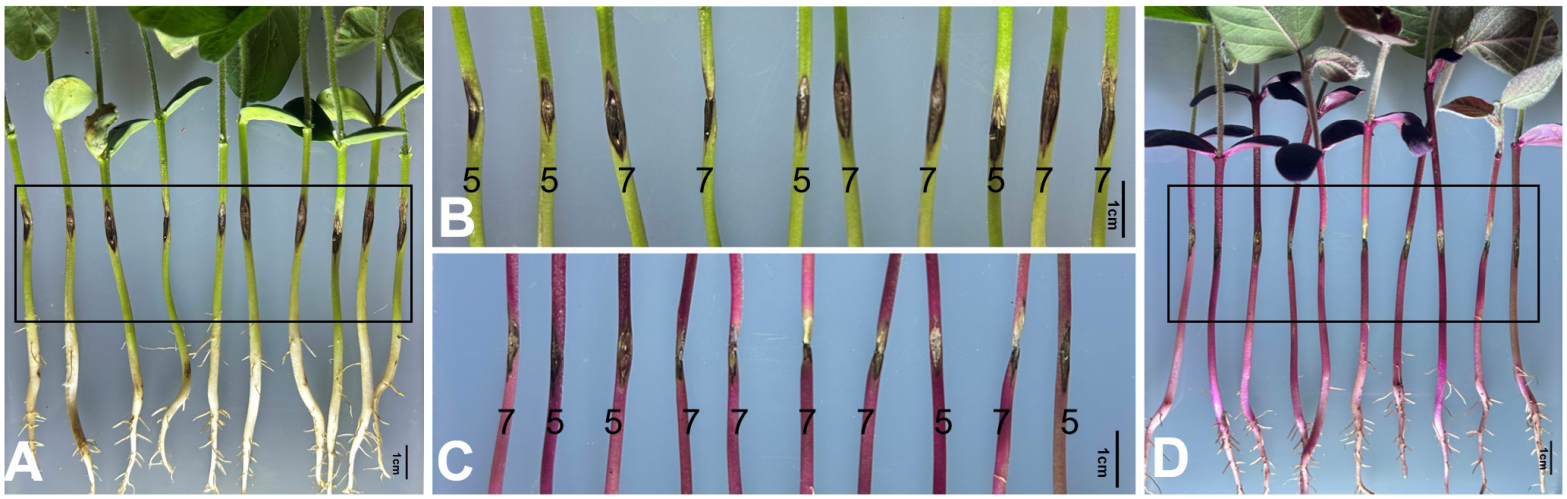
Hypocotyl inoculation through wounding with *P. longicolla* strain CGMCC NO.3.14895. **(A, B)**, wildtype Williams82. **(C, D)**, *RUBY* transgenic line RLine1. The numbers in **(B, C)** are the grading values of individual plant infected by *P. longicolla*. Bars=1cm.

Four days after inoculating the hypocotyls of soybean with *P. longicolla* strain CGMCC NO.3.14895, observation and measurement were conducted on the infection and proliferation of the pathogen at the puncture sites of the hypocotyls. The value of grading standard for individual plant susceptibility was showed in **Figures 5B, C**. The DIs of RLine1 and wildtype Williams82 infected by *P. longicolla* were 68.89% and the resistance grade of both reached high susceptibility (HS). The results showed that, in terms of the morphological characteristics of lesions (including the distribution of browning areas and the hyphal expansion pattern) and the overall disease severity, the inoculated hypocotyl parts of soybean plants rich in betalain were consistent with those of the wildtype plants. Therefore, this study confirms that the accumulation of betalain in plants does not alter the infection of soybean by *P. longicolla* or the expansion characteristics of lesions.

### Effect of *RUBY* transgenic soybean on brown planthopper

**Figure 6:**
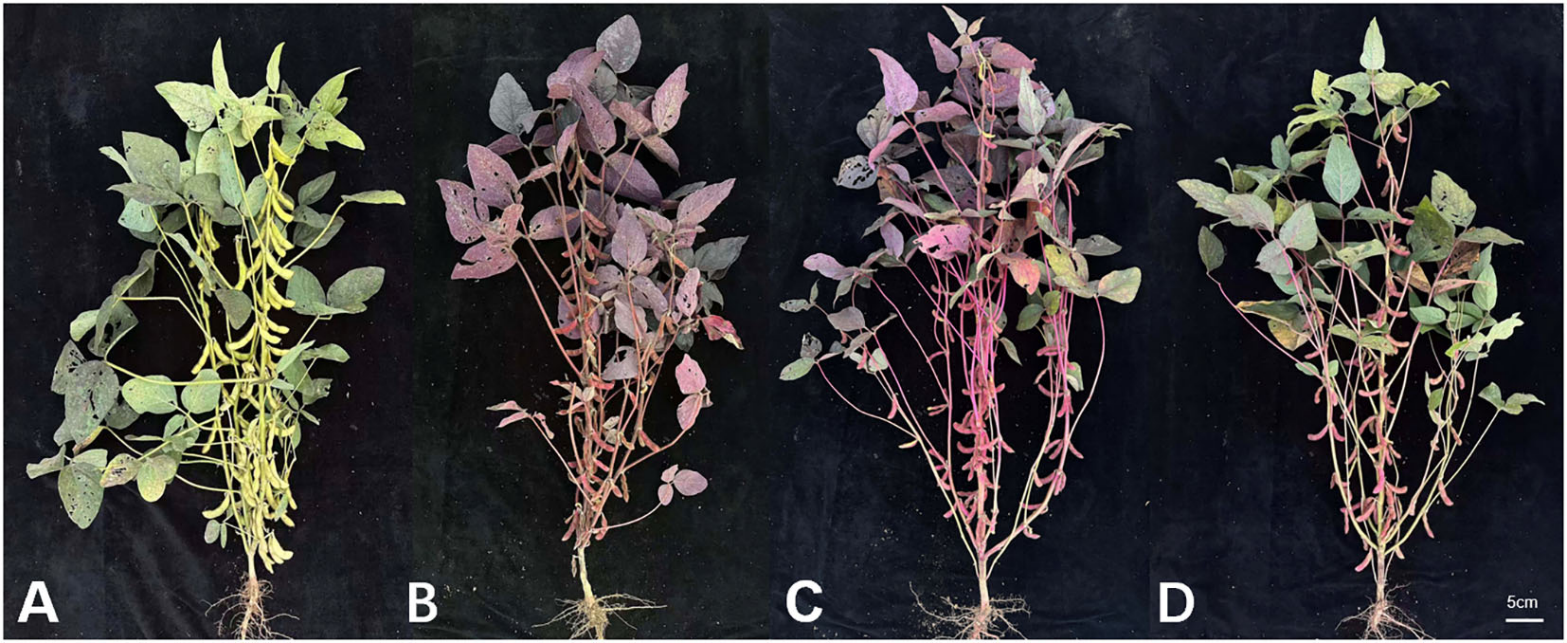
A complete soybean plant at the R7 stage. **(A)** wildtype Williams82**. (B, C, D)** the *RUBY* transgenic positive lines, RLine1, RLine2, and RLine6, respectively. Bars=5cm.

**Figure 7:**
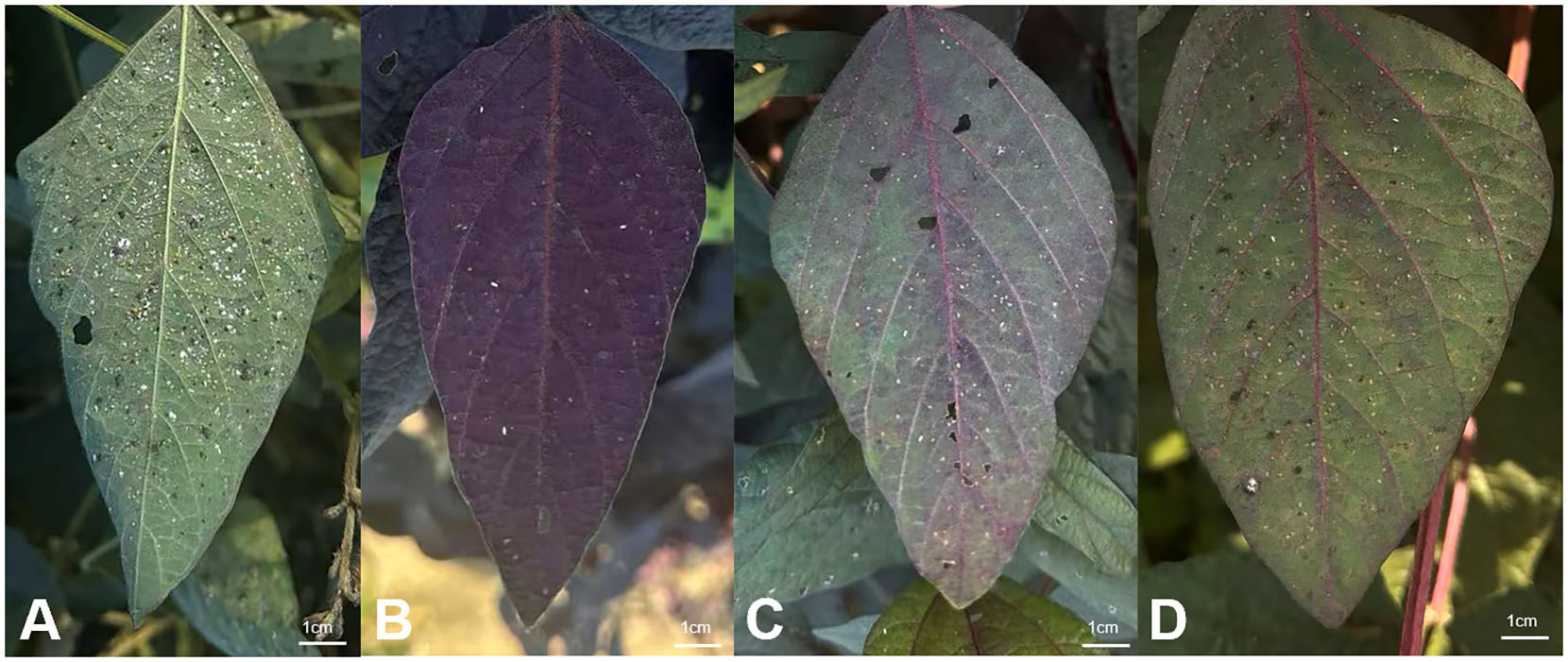
The eggs of the brown planthopper are deposited on the leaves of Williams82 **(A)** and *RUBY* transgenic lines, Rline1 **(B)**, Rline2 **(C)**, Rline6 **(D)**. Bars=1cm.

**Figure 8:**
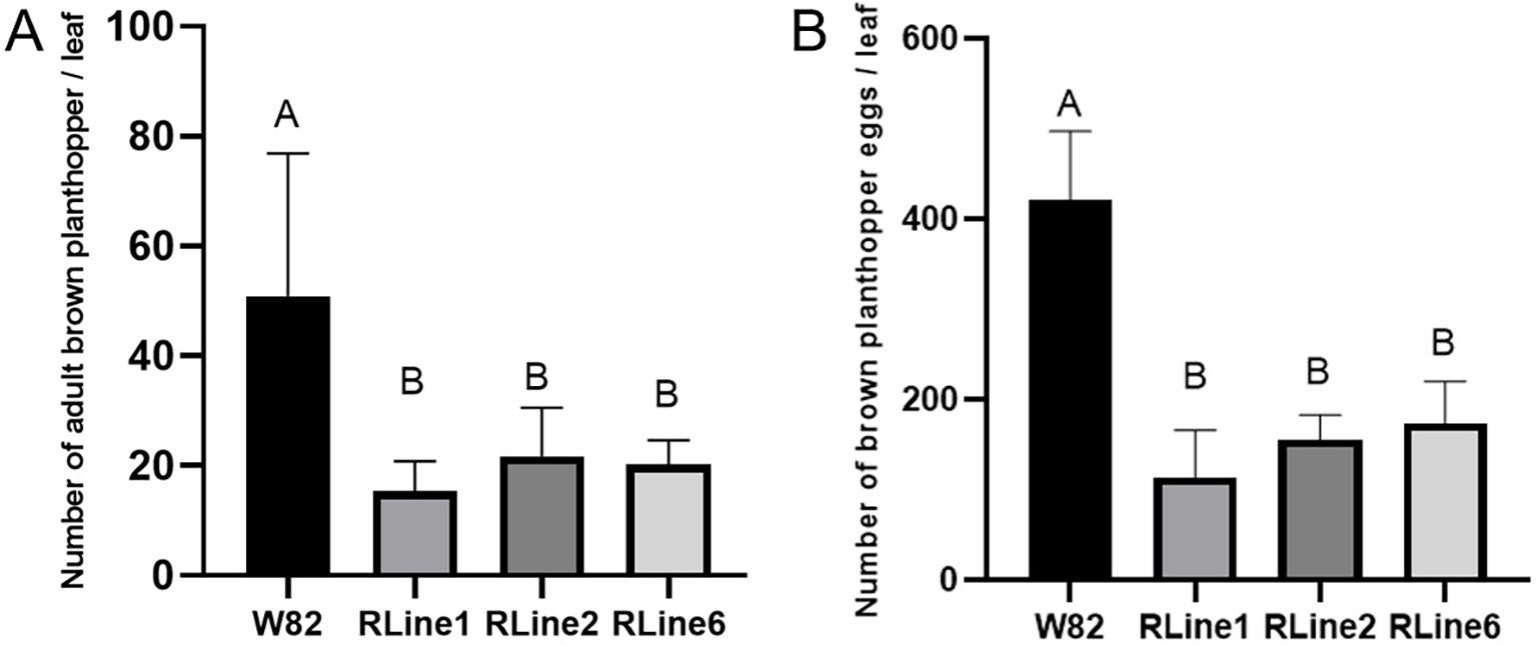
Statistical analysis of the adults **(A)** and eggs **(B)** of brown planthopper on leaves of Williams82 and *RUBY* transgenic lines, Rline1, Rline2, Rline6. Results are the mean ± SD for ten biological replicates. Values followed by the same letter (A or B) indicate no significant difference between them, while those followed by different letters indicate an extremely significant difference, p<0.01 (one-way ANOVA).

In the field cultivation conditions, the three *RUBY* transgenic lines, RLine1, RLine2, and RLine6, exhibited continuous phenotypic differences in betalain accumulation. Based on their coloring intensity, they could be clearly classified into three main types (**Figure 6**): the RLine1 plants showed an entire-plant deep red type, the RLine2 line exhibited a bright red color, leaves of the RLine6 line were mainly green with a light red tint, and their pods are red. No major diseases occurred during the 2025 soybean growing season (July to October, 2025), but insect infestation was severe in the soybean planting field, especially the damage caused by brown planthopper biotype II (*Nilaparvata lugens*). Meanwhile, it was observed that compared with the wildtype Williams82, the number of brown planthoppers on *RUBY* transgenic soybean plants and the number of attached eggs on the abaxial surface of leaves were much lower. The mean number of adult brown planthoppers per leaf reached 50.8 in the wildtype Williams82, whereas the numbers in the three transgenic lines, Rline1, Rline2, and Rline6, were only 15.3, 21.7, and 20.2, respectively. To further verify whether the accumulation of betacyanin in soybean leaves affects the feeding and oviposition of brown planthoppers, six plants each of *RUBY* transgenic lines (RLine1, RLine2, RLine6) and wildtype Williams82 were randomly selected at the soybean (R6, Soybean developmental stage of full pod-filling stage) stage. Photographs were taken while minimizing disturbance to brown planthopper adults to count them. Subsequently, six expanded trifoliate leaves (one per selected plant) were collected, and the number of terminal leaflets of each trifoliate leaf as well as the number of brown planthopper eggs were counted (**Figure 7**). The mean number of eggs per leaf reached 420.5 in the wildtype Williams82, whereas the numbers in the three transgenic lines, Rline1, Rline2, and Rline6, were only 113.7, 155.2, and 173.5, respectively. The statistical results showed that the number of brown planthopper adults and eggs on the leaves of *RUBY* transgenic lines was extremely significantly lower than that on wildtype Williams82 (**Figure 8**). With the fading of red color in leaves, the average number of eggs per leaf gradually increased, but the difference did not reach a statistically significant level (**Figure 8B**).

### Field agronomic traits of *RUBY* transgenic soybeans

**Figure 9:**
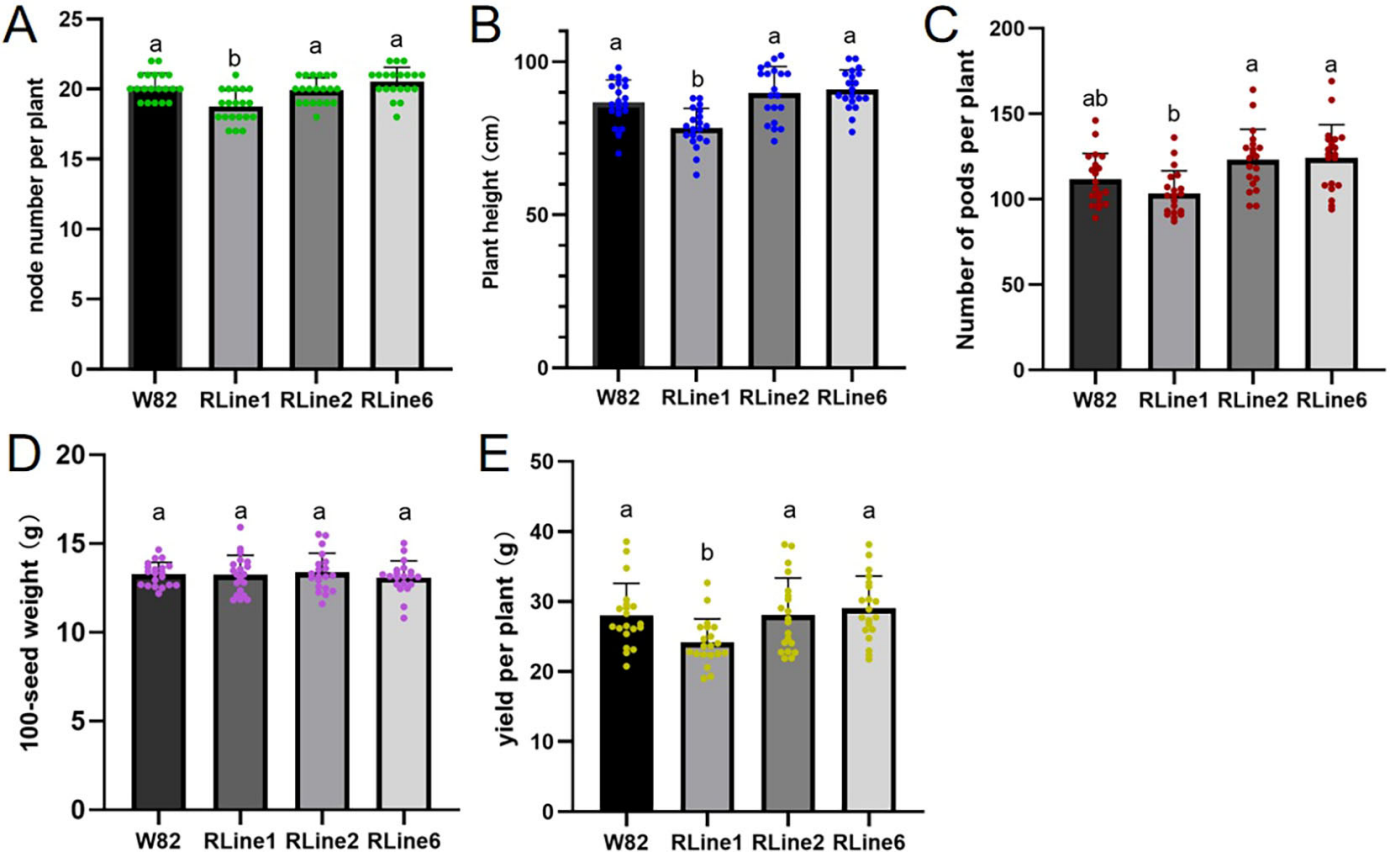
Statistical analysis of node number on main stem **(A)**, plant height **(B)**, pod number per plant **(C)**, 100-seed weight **(D)**, and yield per plant **(E)** in wildtype Williams82 and *RUBY* transgenic lines, Rline1, Rline2, Rline6. Results are the mean ± SD for twenty biological replicates. Values followed by the same lowercase letter (a or b) indicate no significant difference between them, while those followed by different lowercase letters indicate a significant difference, p<0.05 (one-way ANOVA).

To systematically evaluate the effects of betalain accumulation in *RUBY* transgenic soybeans on the main agronomic traits of soybeans in the field, transgenic lines, RLine1, RLine2, RLine6, and the wildtype Williams82 were used as experimental materials. The node number on main stem, plant height, pod number per plant, 100-seed weight, and yield per plant were conducted comparative analyses. The results showed that there were no significant differences in node number on main stem, plant height, and yield per plant between Williams82 and the transgenic lines RLine2 and RLine6. However, these three traits were significantly reduced in RLine1 and showed significant differences compared with Williams82, RLine2, and RLine6 (**Figures 9 A, B, E**). Regarding pod number per plant, no significant difference was observed between the Williams82 and the three transgenic lines. Nevertheless, the pod number of RLine1 was significantly lower than that of RLine2 and RLine6 (**Figure 9 C**). In terms of 100-seed weight, there were no significant differences between the Williams82 and all the transgenic lines (**Figure 9D**). Collectively, these findings indicate that RLine2 and RLine6, which have low betalain content, exerted no adverse effects on various agronomic traits under field experimental conditions. In contrast, RLine1, with high betalain content, exhibited negative impacts on node number on main stem, plant height, and yield.

## Discussion

In molecular biology research and biotechnology applications, marker technology serves as a core tool for tracking gene expression, cellular activities, and transgenic events. Although traditional visual marker technologies, such as GUS (β-glucuronidase), luciferase (LUX), and fluorescent proteins (e.g., RFP, GFP), have been widely applied, they all exhibit significant limitations (Fan et al., 2020). In recent years, the *RUBY* reporter system has been shown to mediate the catalytic synthesis of betalain from tyrosine (He et al., 2020). Importantly, this process is not restricted by species or genotype, giving the *RUBY* system broader application potential. In this study, a total of nine *RUBY* transgenic positive lines with red leaves were obtained. However, the leaves, stems, and other tissues of the T1 generation lines (RLine4, 5, and 7) no longer exhibited red color. We inferred that these three lines were chimeras or transgene silencing. This demonstrates that the visual characteristic of the *RUBY* reporter system provides an excellent platform for studying the phenomenon and mechanism of transgene silencing (Kramer et al., 2025).

Plant secondary metabolites help plants defend against biotic stresses by repelling biotic stress factors, attracting their natural enemies, or exerting toxic effects on them (Al-Khayri et al., 2023). Betalain, as a type of secondary metabolite, possesses notable protective activities, particularly shielding plants from abiotic stresses, diverse pathogens, and viruses (Jin et al., 2025). But it remains unclear whether its accumulation in soybean organs affects the interactions between plants and microorganisms. In this study, we investigated the interactions between soybean and microorganisms, including the symbiotic nitrogen fixation with rhizobia and the antagonistic relationship with pathogenic fungi such as *P. longicolla*. In the symbiotic system of soybean and rhizobia, there were no significant differences in nodule number, fresh weight, and dry weight between the *RUBY* transgenic lines and the control. This indicates that betalain accumulation did not interfere with the infection process of *B. japonicum* on soybean or the nodule formation process. After inoculating transgenic soybean with *P. longicolla*, there were no significant differences in lesion expansion, browning area, or disease susceptibility between the transgenic lines and the wildtype soybean. This suggests that betalain did not alter the invasion and expansion of *P. longicolla* in the host.

Secondary metabolites of plants play a crucial role in defending against herbivorous insects. In this study, field surveys revealed that the number of brown planthopper adults and eggs attached to the leaves of *RUBY* transgenic soybean was significantly lower than that on wildtype soybean. This finding suggests that betalain accumulation may endow soybean with a certain level of insect resistance potential. This phenomenon is consistent with the research results on grain amaranth and quinoa, where the red phenotype, due to its spectral characteristics deviating from the green color preferred by herbivorous insects, may be recognized as an ‘unsuitable’ host (Portillo-Nava et al., 2021). Meanwhile, some reports have shown that betalain accumulation is often accompanied by the upregulation of insect-resistant secondary metabolites (such as ferulic acid and caffeic acid), forming a synergistic defense mechanism (Stintzing et al., 2004; Liu et al., 2025). Although the specific mechanism by which betalains regulate insect behavior still needs in-depth analysis, the findings of this study provide preliminary evidence for the application of betalains in insect-resistant crop breeding. The data from this study remain preliminary, and specific assays targeting feeding and oviposition behaviors are currently lacking, making it difficult to substantiate the proposed mechanism.

The *RUBY* reporter system exhibits multiple advantages in plant breeding and molecular biology research. However, in this study, only one soybean cultivar, one rhizobial strain, one pathogen isolate, and one type of pest insects were employed in one soybean growing season. Therefore, the experimental data of this study have certain limitations, making it difficult to draw the conclusion that betalain accumulation exhibits excellent non-interference in all soybean cultivar–microbe interaction systems. In particular, the insect resistance identification tests of soybeans should be conducted in independent culture chambers to eliminate the influence of weather conditions. Moreover, this study neither strictly distinguished the insect stages nor established rigorous criteria for insect resistance evaluation. Nevertheless, it provides a valuable reference for future research on soybean–microbe interactions.

## Methods

### Construction of the *RUBY* over-expression vector and *Agrobacterium tumefaciens*-mediated transformation

To construct the *RUBY* expression vector for soybean transformation, a pair of PCR primers 35AD1F/35GTR (all the primers’ sequences were listed in **Supplementary Table S1**) was designed for amplifying the *RUBY* expression cassette. PCR amplification was performed using the p35RUBY vector (Lyu et al., 2024) as the template with KOD One PCR Master Mix Blue (TOYOBO, Japan). The PCR product was digested with *Nco*I, and then ligated into the vector pCAMBIA3301 digested with *Nco*I and *Pml*I with T4 ligase, thereby replacing the β-glucuronidase (*GUS*) gene. After the recombined vector was verified to be correct by Sanger sequencing, it was used to transform the soybean cultivar Williams82 via the *A tumefaciens* EHA105 mediated transformation (Olhoft et al., 2003). Glufosinate herbicide (10 mg/L) was added to the selection and meristem culture medium.

After the *RUBY* transgenic T0 lines were planted in the field, self-pollination was conducted to obtain *RUBY* transgenic soybean T1 seeds. Subsequently, the T1 seeds were planted in the field and self-pollinated, followed by the harvest of T2 soybean seeds. The experiments for trait identification of *RUBY* transgenic lines and field phenotype observation were carried out in the field, while the experiments on the interaction with pathogenic bacteria were conducted in the laboratory. Some T0 plants showed a red color (with betalain accumulation), but the red color of their self-pollinated T1 plants disappeared. For the T1 plants of these transgenic lines, transgenic detection was performed using the PCR method to amplify the *Bar* gene with the primer set Bar1/2.

### Interaction between *RUBY* transgenic soybean and rhizobia

To evaluate whether betalain affects the infection and colonization of rhizobia, *Bradyrhizobium japonicum* strain USDA110 was used to inoculate *RUBY* transgenic soybean. The *B. japonicum* USDA110 was preserved by our group (Fan et al., 2020). The *B. japonicum* strain preserved in glycerol was taken out from a -80°C refrigerator, activated on YEM (Yeast Extract Mannitol) solid medium, and cultured at 28°C for 7–10 d. Subsequently, the bacterial cells were rinsed with sterile water to prepare a bacterial suspension, and the concentration of the suspension was adjusted to OD_600_ = 0.15 by detection with a spectrophotometer.

*RUBY* transgenic line RLine1, which was rich in betalain, and wildtype Williams82 were used as experimental materials. The seeds of RLine1 and Williams82, respectively, were first surface-sterilized with 75% ethanol for 1 minute, then rinsed 3 times with sterile water, and sown in sterilized plastic culture boxes (with high-temperature sterilized vermiculite as the substrate). The soybeans were placed in an artificial climate chamber with the photoperiod 16 h light/8 h dark at 26°C. Seven days post-sowing, the seedlings were inoculated with *B. japonicum*. A bacterial suspension was prepared and adjusted to an optical density at 600 nm (OD_600_) of 0.15. Inoculation was performed by root drenching, where 20 mL of the rhizobial suspension was applied directly to the root zone of each seedling. Following inoculation, the plants were maintained under the same growth conditions described above. At 28 days post-inoculation (dpi), the roots of nine soybean seedlings were washed free of substrate. The total number of root nodules per plant was quantified via manual inspection. The surface water of root nodules was blotted dry with absorbent paper, and the fresh weight of soybean root nodules was measured. The root nodules were then placed in an oven at 80°C and dried for 24 hours, followed by the measurement of their dry weight. Data from 10 independent plants were collected separately for Rline1 and the wildtype soybean for statistical analysis.

### Resistance identification of *RUBY* transgenic soybean to *P. longicolla*

To evaluate whether betalain affects the infection and spread of *P. longicolla* in soybean, *RUBY* transgenic soybean plants were inoculated with *P. longicolla* CGMCC NO.3.14895. The *P. longicolla* strain preserved in glycerol was taken out from a -80°C refrigerator and activated on solid PDA medium for 4–5 d at 26°C. Subsequently, the strain was inoculated again on PDA (Potato Dextrose Agar) medium for subculture culture.

Inoculation test of *P. longicolla* was conducted by the hypocotyl wound inoculation method with *RUBY* transgenic line RLine1 and wildtype Williams82 (Zhao et al., 2021). Soybean seeds were sown in vermiculite substrate and cultured on a light incubator shelf with16 h light/8 h dark at 26°C. A puncture needle was used to make a wound on the soybean hypocotyl (2 cm below the cotyledons) of 7-day-old seedlings. A mycelial block of approximately 5 mm × 5 mm was picked and attached to the wound, ensuring that the mycelial surface was in close contact with the wound. Ten Rline1 transgenic plants and the wildtype Williams82, respectively, were inoculated. After inoculation, the wound and the mycelial block were covered with moist vermiculite to maintain humidity, and the disease incidence was investigated 4 d later. The grading standard for individual plant susceptibility after soybean plants are infected by *P. longicolla* is shown in **Supplementary Table S2**. The Disease Index (DI) is used to evaluate the disease resistance ability among different plant lines quantitatively. The calculation formula of DI and the disease ‘Resistance Grade’ are shown in **Supplementary Table S3**.

### Field investigation on insect resistance of *RUBY* transgenic soybean

On June 22, 2025, *RUBY* transgenic soybean lines (RLine1, RLine2, RLine3) and wildtype Williams82 were sown in the experimental field located in Shenxian County, Liaocheng City, Shandong Province (36°N, 115°E). The four soybean lines were planted using single-factor randomized block design with three replications. The row spacing for soybean sowing was 40 cm, and during the seedling stage, the plant spacing was thinned to 10 cm, resulting in a planting density of 250,000 plants per hectare. Except that no insect protection measures were taken after the R1 developmental stage, all other aspects followed the Standard field management practices (Du et al., 2020; Zhu et al., 2025b). During the growth period of soybean, the resistance of *RUBY* transgenic soybean to local diseases and insect pests was investigated. During the peak occurrence period of brown planthopper (August-September 2025), 10 leaves were randomly selected from each of the transgenic lines (RLine1, RLine2, RLine6) and Williams82. The number of the adults and egg grains of brown planthopper on the abaxial surface (underside) of the selected leaves was counted. Statistical analysis was performed using GraphPad Prism 10.4.0 software.

### Investigation of field agronomic traits

To evaluate the various agronomic traits of RUBY transgenic soybeans, we sowed RUBY transgenic soybean lines (RLine1, RLine2, RLine3) and the wildtype line Williams82 in the experimental field located in Shen County, Liaocheng City, Shandong Province (36°N, 115°E) on June 22, 2025. The sowing row spacing of soybeans was set at 40 cm, and the seedling spacing was adjusted to 10 cm at the seedling stage, with a planting density of 250,000 plants per hectare.

Standard field management practices were followed, except for insect protection before the developmental stages R3 (Du et al., 2020; Zhu et al., 2025b). After the soybeans matured and were harvested on October 2, 2025, a survey of agronomic traits was conducted on the RUBY transgenic lines and the wildtype Williams82. For each transgenic line, 20 plants were randomly selected to measure plant height, node number, pod number per plant, 100-seed weight, and Harvest weight per plant. All data were statistically analyzed and visualized into charts using GraphPad Prism 10.4.0 software.

## Data Availability

The original contributions presented in the study are included in the article/**Supplementary Material**. Further inquiries can be directed to the corresponding author.

## Supplementary material

Supplementary File 1 **Tables 1**–**3** and **Supplementary Figures S1**-**S2**.

Supplementary File 2 The row data of **Figure 4** and **Figure 8**.
