# Supplementary material for: Creation and resistance evaluation of a new soybean germplasm rich in betalain
Source: Front Plant Sci. 2026 Jan 19;16:1743684. doi: 10.3389/fpls.2025.1743684 (PMC12861338; doi:10.3389/fpls.2025.1743684)

Supplementary Table S1. All primers used in this study.

| Primers | Sequence (5’-3’) | Used in |
| --- | --- | --- |
| 35AD1F | GTGTGACCATGGATCATGCGACCCTC | To amplify *RUBY* expression cassette |
| 35GTR | TCACTATCACTGGAGGCTTGG |  |
| Bar1 | CCGATGACAGCGACCAC | To amplify *Bar* resistance gene |
| Bar2 | CGGTCTGCACCATCGTC |  |


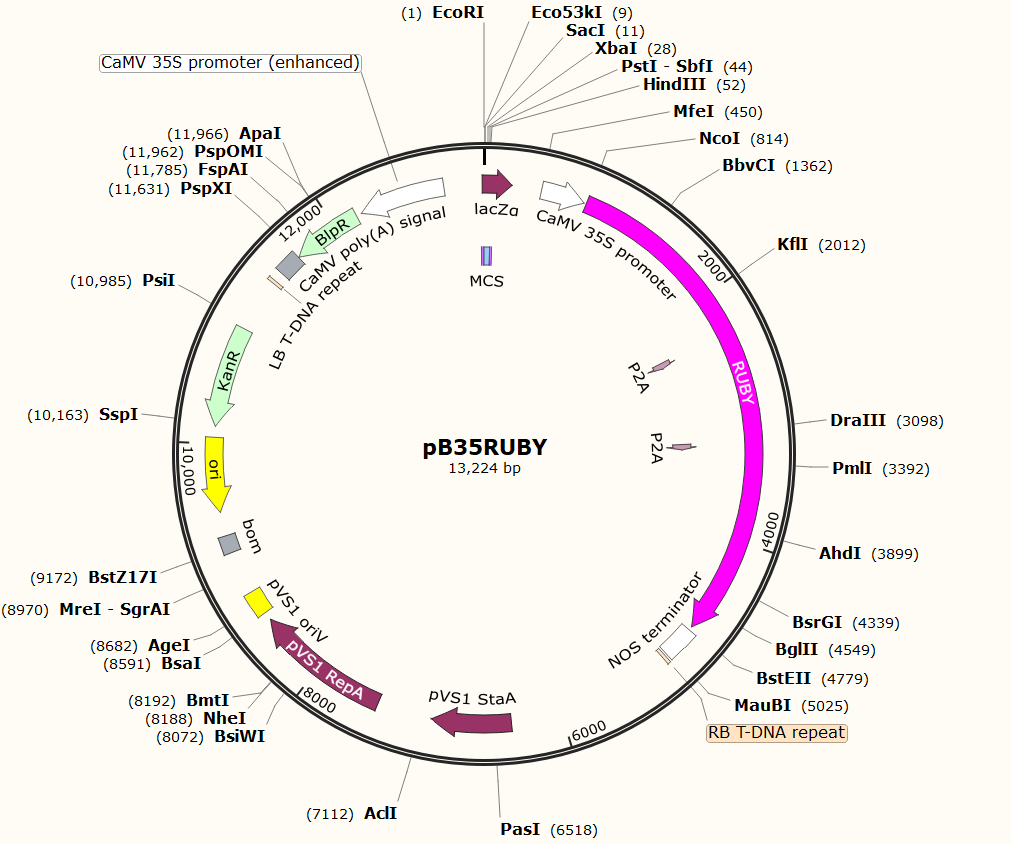


Supplimentary Fig. S1 The map of pB35RUBY vector.


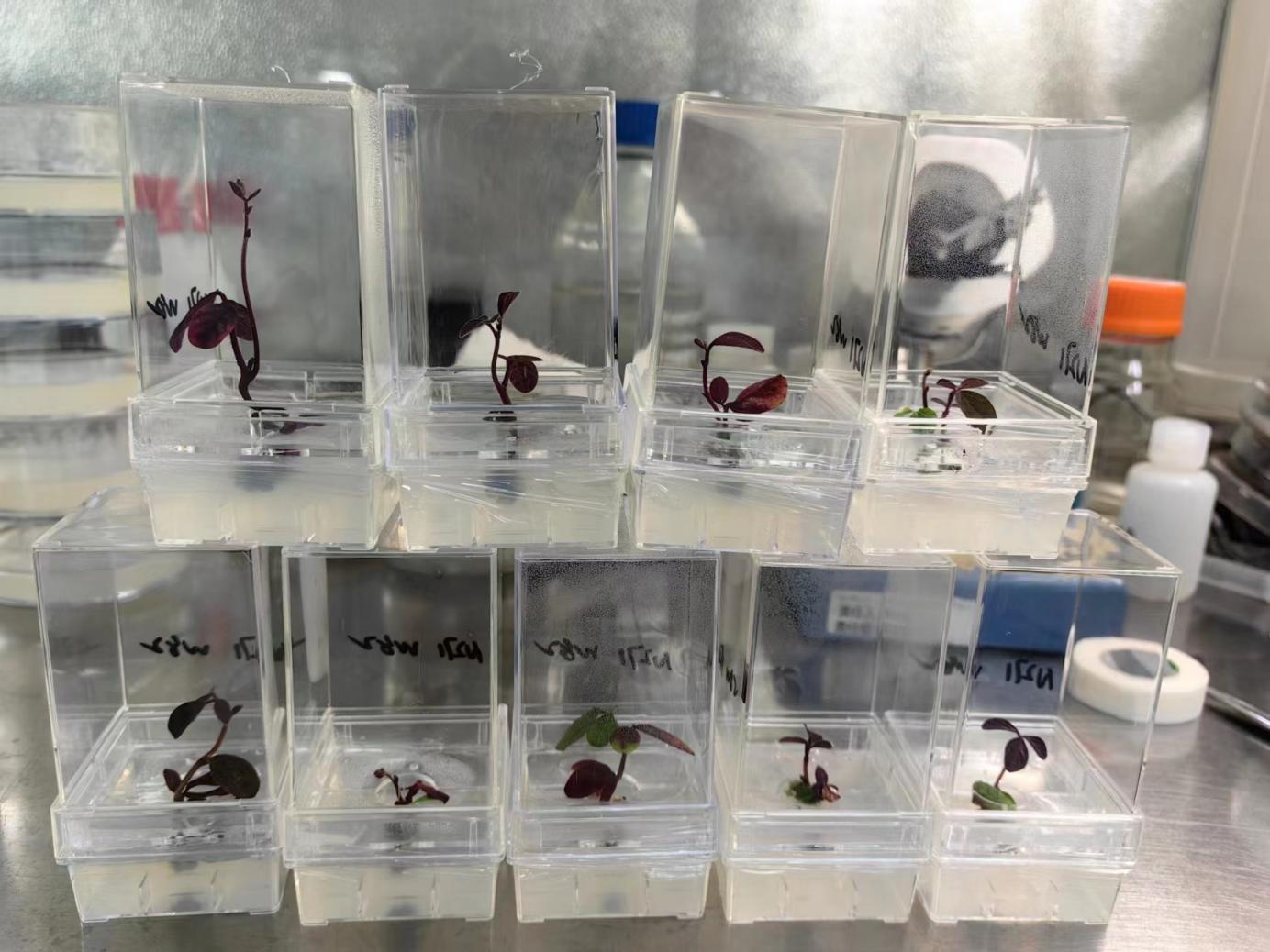


Supplimentary Fig. S2. The total of nine T0 *RUBY* transgenic lines.

Supplementary Table S2. The grading standard for individual plant susceptibility after soybean plant is infected by *P. longicolla*.

| Value | Individual Plant Identification Standard |
| --- | --- |
| 0 | Asymptomatic, or only green or brown lesions appearing at the inoculation site. |
| 1 | Lesions at the inoculation site expand, with an expansion length of approximately 3 mm. |
| 3 | Lesion length at the inoculation site reaches 3–5 mm length. |
| 5 | Lesion length at the inoculation site reaches 5–10 mm length, but no circumferential expansion. |
| 7 | The lesions at the inoculation site have spread to encircle the entire hypocotyl, but the hypocotyl segment has not rotted. |
| 9 | The lesions at the inoculation site encircle the entire hypocotyl, and the hypocotyl rots and breaks, leading to the death of the plant. |

Supplementary Table S3. The calculation formula of DI and the disease resistance grading after soybean plant is infected by *P. longicolla*.

| Resistance Grade | Abbreviation | Range of Disease Index (DI) | Meaning |
| --- | --- | --- | --- |
| Immune | IM | DI = 0 | Completely no disease (no any disease symptoms) |
| High Resistance | HR | 0 < DI ≤ 10% | Extremely mild disease, only a few individuals  have mild disease, and there is almost no impact  on growth or yield |
| Moderate Resistance | MR | 10% < DI ≤ 30% | Relatively mild disease, some individuals are  diseased but the degree is limited, and the impact on growth or yield is minor |
| Moderate Susceptibility | MS | 30% < DI ≤ 50% | Moderate disease, quite a number of individuals  are diseased with moderate degree, and there is  a certain impact on growth or yield |
| High Susceptibility | HS | DI > 50% | Severe disease, most individuals are diseased with high degree, and the impact on growth or yield is significant |


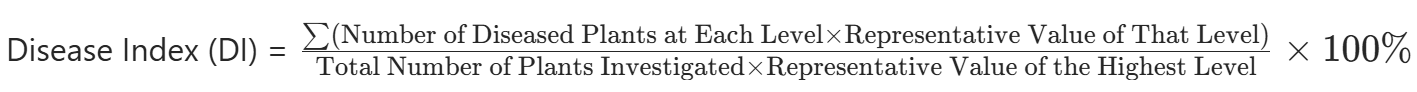

Supplement: Supplementary File 2 — The row data of Figure 4 and Figure 8. [file DataSheet1.docx]
